# Plant-Derived Chimeric Virus Particles for the Diagnosis of Primary Sjögren Syndrome

**DOI:** 10.3389/fpls.2015.01080

**Published:** 2015-12-01

**Authors:** Elisa Tinazzi, Matilde Merlin, Caterina Bason, Ruggero Beri, Roberta Zampieri, Chiara Lico, Elena Bartoloni, Antonio Puccetti, Claudio Lunardi, Mario Pezzotti, Linda Avesani

**Affiliations:** ^1^Department of Medicine, University of VeronaVerona, Italy; ^2^Department of Biotechnology, University of VeronaVerona, Italy; ^3^UTBIORAD-FARM, Laboratory of Biotechnology, ENEARome, Italy; ^4^Rheumatology Unit, Department of Medicine, University of PerugiaPerugia, Italy; ^5^Ospedale Bambin GesùRome, Italy

**Keywords:** molecular farming, Sjögren’s syndrome, diagnosis, ELISA, VPN, PVX, CVP, lipocalin

## Abstract

Plants are ideal for the production of protein-based nanomaterials because they synthesize and assemble complex multimeric proteins that cannot be expressed efficiently using other platforms. Plant viruses can be thought of as self-replicating proteinaceous nanomaterials generally stable and easily produced in high titers. We used *Potato virus X* (PVX), chimeric virus particles, and *Cowpea mosaic virus*, empty virus-like particles to display a linear peptide (lipo) derived from human lipocalin, which is immunodominant in Sjögren’s syndrome (SjS) and is thus recognized by autoantibodies in SjS patient serum. These virus-derived nanoparticles were thus used to develop a diagnostic assay for SjS based on a direct enzyme linked immunosorbent assay format. We found that PVX-lipo formulations were more sensitive than the chemically synthesized immunodominant peptide and equally specific when used to distinguish between healthy individuals and SjS patients. Our novel assay therefore allows the diagnosis of SjS using a simple, low-invasive serum test, contrasting with the invasive labial biopsy required for current tests. Our results demonstrate that nanomaterials based on plant viruses can be used as diagnostic reagents for SjS, and could also be developed for the diagnosis of other diseases.

## Introduction

Sjögren’s syndrome (SjS) is a complex chronic systemic autoimmune disease that may present alone as primary SjS (pSjS) or associated with other autoimmune diseases as secondary SjS (sSjS). The prevalence of pSjS in the general population is between 0.01 and 0.1% with a higher incidence of the disease in women than in men (9:1; Maldini et al., 2013). The clinical symptoms of pSjS include local exocrinopathy, resulting in dry eyes and mouth, extraglandular and systemic manifestations such as arthralgias and fatigue, and severe systemic symptoms such as vasculitis, pulmonary fibrosis, glomerulonephritis and neurological pathology (Tzioufas et al., 2012). The heterogeneity of pSjS often delays diagnosis (Patel and Shahane, 2014) and many cases are overlooked due to the absence of sensitive and specific disease markers and other definitive criteria for classification (Plesivcnik Novljan et al., 2014; Rasmussen et al., 2014).

Current diagnostic criteria include the presence of antinuclear, anti-Ro-SSA and/or anti-La-SSB antibodies, but antinuclear antibodies are only detected in 60–85% of patients, anti-Ro-SSA antibodies in 52–67% of patients and anti-La-SSB antibodies in ∼49% of patients (Nardi et al., 2006; Routsias and Tzioufas, 2007; Suresh et al., 2015). The American European Consensus Group (AECG) criteria have been widely adopted and comprise six key signs (Baldini et al., 2012): 1 – ocular symptoms, 2 – oral symptoms, 3 – a positive Schirmer’s test, 4 – a positive labial biopsy, 5 – salivary flow symptoms, and 6 – the presence of autoantibodies anti-SSA (Ro) or anti-SSB (La). At least three of these signs should be present for a preliminary diagnosis of pSjS. If four symptoms are present including 4 and/or 6, or if three of symptoms 3, 4, 5, and 6 are present, then the diagnosis is confirmed. The anti-SSA (Ro) and anti-SSB (La) autoantibodies listed among the AECG criteria are common in other autoimmune diseases and therefore do not offer a rigorous pSjS diagnosis at an early stage when other symptoms are absent (Shen et al., 2012). A labial biopsy is therefore mandatory for confirmation, and is the only indicative diagnosis in the absence of autoantibodies and other symptoms (Patel and Shahane, 2014). This has driven researchers to seek more reliable autoantigens and autoantibodies suitable for early diagnosis.

The expansion of proteomics in medicine has led to the discovery of specific immunodominant epitopes associated with autoimmune diseases, resulting in novel immunoassays with better predictive power. The most widely used immunoassays are based on the enzyme linked immunosorbent assay (ELISA), in which a passively adsorbed antigen is used to identify samples containing reactive antibodies. This is a simple and rapid technique which is routinely applied in many laboratories (Engvall and Perlmann, 1972). ELISAs tend to be highly specific, and where cross-reactivity results in a false-positive diagnosis, this is usually less to do with multiple antibody types in polyclonal sera and more to do with the conservation of epitopes between target and non-target proteins. Whole proteins present several epitopes that increase the likelihood of cross-reactions, so the use of antigenic peptides comprising single epitopes can remove this risk (Haist et al., 1992). For example, the diagnostic peptide ELISA for Chagas disease has a sensitivity of 96.8–100% and a specificity of ∼99% (da Silveira et al., 2001). Peptides are also advantageous because they can be usually chemically synthesized whereas whole proteins must be produced using recombinant DNA technology (Plesivcnik Novljan et al., 2014).

One major drawback of peptide ELISAs is the inefficient binding of peptides to solid substrates such as microtiter plates (Griesmann et al., 1991). Binding depends on the mass of the peptide and its amino acid composition, and if much of the peptide is involved in non-specific absorption then the relevant epitopes can be obscured (Sallberg et al., 1995; Gregorius et al., 1999; Gregorius and Theisen, 2001). Two major strategies have been developed to stabilize the peptides used in diagnostic ELISAs, one based on chemical modification and the other based on the use of biological nanoparticles for peptide display. Whereas chemical modification involves the addition of functional groups that bind to polystyrene (Gregorius and Theisen, 2001; Kumada et al., 2009), the nanoparticle-based method involves the use of virus-derived nanoparticles (VNPs) as scaffolds allowing the peptide to be presented and accessed (Sanchez et al., 2013). Structural evaluations should be carefully considered in the context of VNPs conformation and peptide displaying region. Plants can be used successfully to produce plant VNPs in large quantities. These VNPs can be used to coat ELISA plates, resulting in a substantial improvement in the detection of specific antibodies in sera compared to peptide ELISAs (Sanchez et al., 2013). VNPs displaying antigens have also been developed as vaccines, imaging reagents and drug delivery vehicles (Lico et al., 2012). Several plant viruses have been exploited for these purposes including *Cowpea mosaic virus* (CPMV; Montague et al., 2011), *Tomato bushy stunt virus* (Kumar et al., 2009), *Tobacco mosaic virus* (McCormick and Palmer, 2008), *Turnip mosaic virus* (Sanchez et al., 2013), and *Potato virus X* (PVX; Lico et al., 2009).

Virus-derived nanoparticles displaying pSjS-associated peptides have the potential to enhance the sensitivity and specificity of autoantibody detection and therefore allow earlier and more accurate diagnosis. Autoantibodies have been detected against several candidate proteins in pSjS animal models, including salivary gland protein-1 (SP1), carbonic anhydrase 6 (CA6), and parotid secretory protein (PSP), but these have proven unsuitable for diagnosis in humans (Shen et al., 2014). In contrast, lipocalin is selectively recognized by sera from human pSjS patients and could therefore provide a suitable autoantigen for diagnostic ELISAs (Johnsson et al., 2003; Navone et al., 2005).

Tear lipocalin is a protein belonging to the lipocalin family and the calycin superfamily, which are a diverse set of proteins that function as extracellular binding proteins. Lipocalins are a family of low molecular weight proteins (18–40 kDa) with prevalent extracellular functions. Specifically tear lipocalin is highly expressed both in tears and saliva and it accounts for about 15–33% of the protein tear and it is the major lipid binding protein in human tear (Dartt, 2011). Together with other protein of lipocalins family, tear lipocalin has been termed immunocalins, related to their role in immunity. Particularly tear lipocalins could have protective immunoregulatory, anti-inflammatory, and antimicrobial effects in the tears and ocular surface and together with the other immunocalins seems to act as part of the cytokine immune network and as a key regulators of inflammatory cells, included natural killer, neutrophils, monocytes, macrophages, B and T lymphocytes and interfering with platelet aggregation and adherence of neutrophils and monocytes to vascular endothelium (Gasymov et al., 2005).

Given the reported relevance of lipocalin in SjS pathogenesis, we produced VNPs based on CPMV and PVX displaying the immunodominant lipo peptide from lipocalin (Navone et al., 2005) and compared their sensitivity, specificity, reproducibility and stability in diagnostic ELISAs compared to the ELISAs based on the synthetic peptide. The use of PVX- and CPMV-based systems, characterized by different viral shapes and peptide display context, allows to evaluate the influence of VNP structures on ELISA performance.

## Materials and Methods

### Ethical Statement

A written informed consent was obtained from all the participants in the study. The study was approved by the local Ethical Committee (University of Verona) and all clinical investigations have been conducted according to the principles expressed in the Helsinki declaration.

### Patients and Controls

Between January 2005 and December 2013, we obtained serum samples from patients and healthy controls. Blood samples were collected from the participants using a Vacutainer system (Becton Dickinson, Franklin Lakes, NJ, USA) and were stored at –20°C. All the sera were tested for antibodies against nuclear antigens (ANA), extractable nuclear antigens (ENA), and lipocalin by ELISA. Blood samples were obtained after all the subjects provided written informed consent.

We studied a cohort of 91 patients (5 males and 86 females, age range 28–73 years) affected by pSjS (Vitali et al., 2002), attending the Unit of Autoimmune Diseases at the University Hospital of Verona. A cohort of 60 patients affected by rheumatoid arthritis (RA), systemic sclerosis (SSc), and systemic lupus erythematosus (SLE) was also studied as disease controls. RA patients met the American College of Rheumatology classification criteria (Aletaha et al., 2010), the SSc patients met the American College of Rheumatism/European League Against Rheumatism criteria (van den Hoogen et al., 2013a,b) and the SLE patients met the American College of Rheumatology and Systemic Lupus International Collaborating Clinics criteria (Yu et al., 2014; Amezcua-Guerra et al., 2015). All the patients were enrolled consecutively regardless of disease activity and treatment, and were assessed for clinical features and organ damage. A further cohort of age/sex-matched healthy subjects served as a control group.

### Lipocalin Peptide Synthesis

The lipocalin synthetic peptide FEKAAGARGLST (lipo) was designed based on the sequence of tear lipocalin as previously described (Navone et al., 2005) and purchased with free amino- and carboxy- terminal ends by Tib MolBiol (Genoa, Italy). In particular, lipocalin peptide is part of the whole protein corresponding to the sequence spanning from 148 to 159 aminoacids of the whole protein (UniProtKB/Swiss-Prot: Q5VSP4.1). The purity of the peptide was 98%.

### PVX Chimeric Virus Particles

Chimeric PVX particles displaying the lipocalin peptide (PVX-lipo) were prepared using the pPVX*Sma* vector and cloning procedure as previously described (Lico et al., 2006). A sense and antisense oligonucleotide pair was designed to amplify a product with NheI and SmaI compatible ends in which the lipocalin peptide sequence optimized for *Nicotiana benthamiana* codon usage was preceded by an ATG codon and a serine residue (5′-CTA GCC TCG AGA TGT CTT TTG AAA AGG CTG CTG GTG CTA GAG GTT TGT CTA CTC-3′ and 5′-CCG GGA GTA GAC AAA CCT CTA GCA CCA GCA GCC TTT TCA AAA GAC ATC TCG AGG-3′). The final expression vector (pPVX*Sma*-lipo) was used for the primary infection of 4–5-week-old *N. benthamiana* plants, by abrading the surface of two leaves with 20 μg of plasmid DNA and carborundum (VWR International, Milan, Italy). The expression of the chimeric coat protein (CP) gene and the genetic stability of viral genome after several re-infection cycles (using sap from symptomatic leaves in the previous infection cycle) were verified by RT-PCR (Lico et al., 2006). The presence of PVX particles in infected leaf protein extracts was confirmed by western blot analysis. Briefly, plant tissue was ground to a fine powder under liquid nitrogen and homogenized in three volumes of phosphate buffered saline (PBS) containing protease inhibitor cocktail (Sigma–Aldrich, St Louis, MO, USA). After centrifugation (30,000 × *g*, 30 min, 4°C), the supernatant was collected and the total soluble protein (TSP) extracts were separated by 14% sodium dodecylsulfate polyacrylamide gel electrophoresis (SDS-PAGE) and transferred onto a nitrocellulose blotting membrane (GE Healthcare, Uppsala, Sweden). The membrane was probed for 2 h at room temperature using the alkaline phosphatase-conjugated anti-PVX CP antibody (Agdia, Elkhart, IN, USA) diluted 1:200, followed by detection using NBT/BCIP (Sigma–Aldrich, St Louis, MO, USA).

For the large-scale purification of PVX-lipo, approximately 50 g of symptomatic *N. benthamiana* leaves was processed as previously described (Uhde et al., 2005). The purity of the particles was verified by 14% SDS-PAGE and silver staining, and the concentration was determined by measuring the absorbance at 280 nm. *N. benthamiana* plants were also infected with pPVX201 (Baulcombe et al., 1995) for the production and purification of unmodified PVX particles using the same procedure.

### CPMV Empty Virus Like Particles

Cowpea mosaic virus empty virus-like particles (eVLPs) displaying the lipocalin peptide (CPMV-lipo) were produced using the vectors pEAQ-*HT*-VP60 and pEAQ-*HT*-24K (Saunders et al., 2009). A sense and antisense oligonucleotide pair was designed to amplify a product with NheI and AatII compatible ends (5′-CTA GCA CTC CTC CTG CTT TTG AAA AGG CTG CTG GTG CTA GAG GTT TGT CTA CTC CAT TTT CAG ACG T-3′ and 5′-CTG AAA ATG GAG TAG ACA AAC CTC TAG CAC CAG CAG CCT TTT CAA AAG CAG GAG GAG TG-3′) which was inserted into pEAQ-*HT*-VP60 digested with the same enzymes to yield pEAQ-*HT*-VP60-lipo. *Agrobacterium tumefaciens* strain LBA4404 was transformed with pEAQ-*HT*-VP60-lipo or separately with pEAQ-*HT*-24K by electroporation, and bacterial cultures carrying each vector were grown separately to the stable phase in Luria Broth (LB medium) supplemented with the appropriate antibiotics. The cultures were then centrifuged at 4000 × *g* and resuspended in MMA buffer (10 mM MES pH 5.6, 10 mM MgCl_2_, 100 mM acetosyringone) to an OD_600_ of 0.8. After incubation for 1–4 h at room temperature, equal volumes of the two bacterial suspensions were mixed and used for the syringe infiltration of 4–5-week-old *N. benthamiana* leaves (four expanded leaves per plant). Infiltrated leaves were sampled 6 days post infection (dpi). TSP was extracted from infiltrated leaves and analyzed by western blot to confirm the presence of eVLPs (Saunders et al., 2009). The same procedure was followed for the expression of unmodified CPMV eVLPs, by using pEAQ-*HT*-VP60 instead of pEAQ-*HT*-VP60-lipo.

For the large-scale purification of CPMV-lipo, approximately 30 g of infiltrated leaves were homogenized in a blender with four volumes (∼120 ml) of 0.1 M sodium phosphate buffer (pH 7.0) supplemented with 2% (w/v) polyvinylpolypyrrolidone and complete EDTA-free protease inhibitor cocktail (Roche, Basel, Switzerland). The extract was clarified by filtration through two layers of Miracloth (Merck Millipore, Darmstadt, Germany) and centrifugation (30,000 × *g*, 1 h, 4°C). The supernatant was fractionated by anionic exchange chromatography (DEAE Sephadex A-50; GE Healthcare, Uppsala, Sweden), with a sample-to-resin ratio of 4:1. The flow-through fraction was concentrated to 4 ml, centrifuged to remove insoluble particles (10,000 × *g*, 10 min, 4°C) and purified by size-exclusion chromatography (HiPrep 16/60 Sephacryl S-500 HR; GE Healthcare, Uppsala, Sweden) in 0.1 M sodium phosphate buffer (pH 7.0) supplemented with 0.15 M NaCl at a flow rate of 0.8 ml/min. Eluted fractions were analyzed by standard 12% SDS-PAGE and silver staining. Fractions containing CPMV eVLPs were pooled and concentrated using a 100 kDa cut-off centrifugal filter (Merck Millipore, Darmstadt, Germany). Purified particles were analyzed by 12% SDS-PAGE and silver staining, and were quantified by measuring the absorbance at 280 nm.

### Nanoparticle Characterization

Purified PVX particles were distributed on carbon/formvar film-coated 400 mesh copper grids (Electron Microscopy Sciences, Hatfield, PA, USA) and stained with 2% (w/v) uranyl acetate for analysis using a JEM 1200 EXII (Jeol, Tokyo, Japan) transmission electron microscope. The images were acquired using an SIS Veleta charge-coupled device camera (Olympus, Hamburg, Germany) at the Interdepartmental Center of Electron Microscopy (University of Tuscia, Italy).

Dynamic light scattering (DLS) was carried out using a Zetasizer Nano ZS (Malvern Instruments, Malvern, UK). Purified CPMV eVLPs (0.5 mg/ml) were centrifuged (10,000 × *g*, 10 min, 4°C) before analysis to remove insoluble material. Measurements were taken every 10 s and 12 measurements were averaged from three runs at 25°C.

### ELISA

Maxisorp polystyrene plates (NUNC, Roskilde, Denmark) were coated with the synthetic lipocalin peptide, PVX-lipo, CPMV-lipo or the unmodified VNPs as controls each at a concentration of 40 μg/ml in PBS. The plates were blocked for 1 h with 3% bovine serum albumin (BSA) in PBS and then incubated with diluted serum (1:200) from the patients and controls enrolled in the study overnight at 4°C. The plates were then washed once with 1% Tween-20 in PBS and twice with PBS, before incubating for 3 h at room temperature with an alkaline phosphatase-conjugated anti-human IgG (Sigma–Aldrich, St Louis, MO, USA) in diluting buffer, following manufacturer’s instructions. After three washes, the enzymatic activity was measured using *p*-nitrophenyl phosphate (Sigma–Aldrich, St Louis, MO, USA) and a microplate absorbance reader (Sunrise^TM^ III, Tecan, Männedorf, CH) set at 405 nm.

Optical density values higher than the mean of the control group +3 SD were considered positive.

We used as internal positive control the serum of one SjS patient which was previously used by Navone et al. (2005) to assess the ELISA test with the synthetic lipocalin peptide. Moreover, in the ELISA test with synthetic peptide we used an uncoated well as corrector factor. Similarly, the results for the VNPs displaying the lipo peptide were corrected for the corresponding empty scaffolds.

The sensitivity of the ELISA was calculated as the ratio of test-positive SjS patients to the total number of patients affected by the disease and specificity as the ratio of test-negative individuals to the total of SjS non-affected individuals.

The stability of the ELISA was determined by coating the plates with VNPs as above and then storing at 4°C for 1, 15, 30, or 60 days before testing.

### Statistical Analysis

Quantitative data with a normal distribution were expressed as means ± SD and were analyzed using Student’s *t*-test. A multiple logistic regression analysis simultaneously controlling for the titer of ANA, anti-ENA and anti-lipocalin antibodies, as well as the presence or absence of other symptoms, was used to evaluate the independent association between these variables and pSjS. The specificity and sensitivity of the ELISA was considered by testing its ability to distinguish pSjS patients from healthy controls and patients with other autoimmune diseases. All statistical calculations were carried out using SPSS v21 with *p* < 0.05 considered statistically significant.

## Results

### Segregation of Enrolled Patients and Controls

We enrolled 91 patients affected by pSjS, with a majority of female enrollees (86 female, 5 male) reflecting the sex-based prevalence of the disease. A control group of 120 subjects was also enrolled, comprising 60 age/sex-matched healthy donors and 60 patients affected by other autoimmune disorders, i.e., 20 each with SSc, RA, and SLE. The alternative autoimmune disease groups were screened to exclude those showing evidence of sSjS. The SSc and SLE patients showed ANA titers with a typical pattern specific for each disease. The pSjS patients were assigned to groups according to ANA titer and organ involvement, including 25 patients who were ANA negative but showed histological evidence of salivary gland involvement. Pulmonary and cardiac involvement, such as vasculitis, arthralgias/arthritis and myalgias, were also considered as clinical aspects of interest. The clinical data are summarized in **Table 1**.

**Table 1:** Clinical features and laboratory characterization of pSjS patients and controls, including healthy donors and patients with other autoimmune diseases.

	pSjS subjects (*n* = 91)	Healthy controls (*n* = 60)	SSc patients (*n* = 20)	RA patients (*n* = 20)	SLE patients (*n* = 20)
Sex (female/male)	86/5	57/3	19/1	19/1	20/0
ANA titer	66/91	1/60	20/20	2/20	20/20
Presence SSA/SSB	27/91	0/60	1/20	0/20	3/20
Arthritis/arthralgias	79/91	0/60	1/20	20/20	11/20
Myalgias	58/91	0/60	4/20	0/20	8/20
Pulmonary involvement	3/91	0/60	9/20	2/20	0/20
CNS involvement	1/91	0/60	0/20	0/20	2/20
Vasculitis	3/91	0/60	0/20	0/20	0/20

### Production of PVX and CPMV VNPs as Scaffolds for the Lipocalin Peptide

**FIGURE 1:**
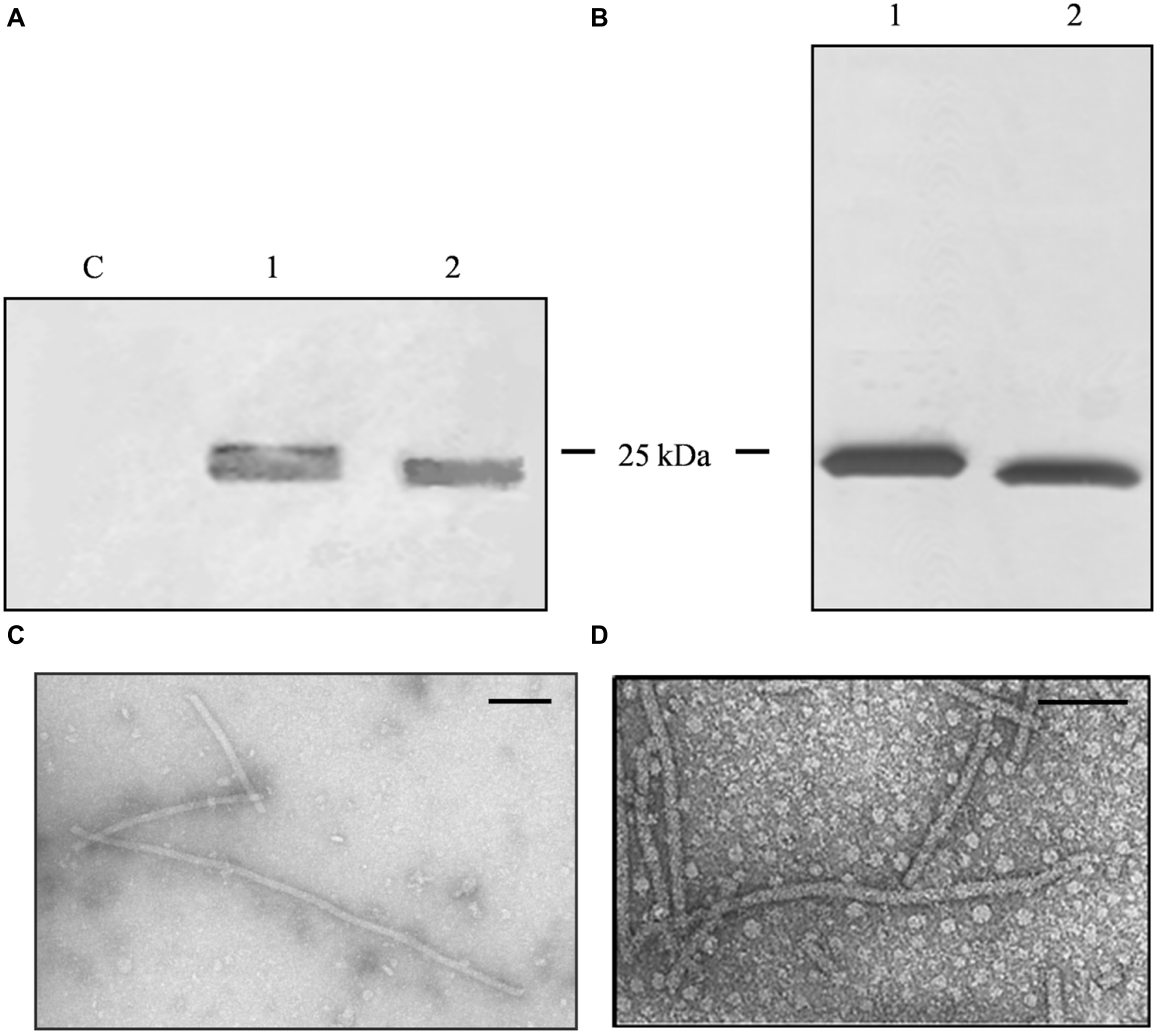
**Analysis of PVX nanoparticles. (A)** Western blot of *N. benthamiana* leaf TSP extracts, 24 μl per lane separated by SDS-PAGE and detected with an alkaline phosphatase conjugated anti-PVX CP antibody. C = non-infected leaf extract, 1 = PVX-lipo leaf extract, and 2 = PVX control leaf extract. **(B)** Silver staining of 750 ng purified particles separated by SDS-PAGE. 1 = PVX-lipo particles and 2 = PVX control particles. Arrows indicate the position of the 25 kDa molecular marker. **(C)** Transmission electron micrographs of purified PVX-lipo particles and **(D)** PVX control particles. Scale bar = 100 nm.

The PVX-lipo VNPs were produced by introducing the lipocalin synthetic peptide sequence into the vector pPVX*Sma* (Lico et al., 2006) to yield pPVX*Sma*-lipo particles in which the peptide was fused to the N-terminus of a truncated CP and displayed on the particle surface. The peptide sequence was optimized for *N. benthamiana* codon usage, and virus movement was promoted by adding a serine residue between the initiator codon and the first native residue (Betti et al., 2012). The production of chimeric PVX particles depends on virus replication, so the genetic stability of the virus genome was verified after several re-infection cycles. The plants were infected using pPVX*Sma*-lipo plasmid DNA to initiate the first cycle, followed thereafter by infection using sap from symptomatic leaves obtained 12 days after each round of infection. The chimeric CP gene remained stable through several passages as demonstrated by RT-PCR and sequencing (data not shown). The presence of PVX-lipo and wild-type PVX was verified by western blot analysis of infected leaf extracts (**Figure 1A**), where it is evident a single band corresponding to the viral CP, with a molecular weight shift in the PVX-lipo sample in comparison to wild-type particles, corresponding to the lipo peptide fused to the CP.

A larger number of plants were infected to obtain sufficient leaf material for particle purification (Uhde et al., 2005). We achieved a yield of 0.11 mg PVX-lipo per gram fresh leaf weight (FLW) and the purity of the particles was verified by SDS-PAGE and silver staining (**Figure 1B**). *N. benthamiana* plants were also infected with pPVX201 to obtain leaf material for the production of unmodified PVX control particles (**Figures 1A,B**). The yield was 0.62 mg/g FLW. Transmission electron microscopy confirmed there were no morphological differences between the chimeric PVX-lipo particles and the control particles (**Figures 1C,D**).

**FIGURE 2:**
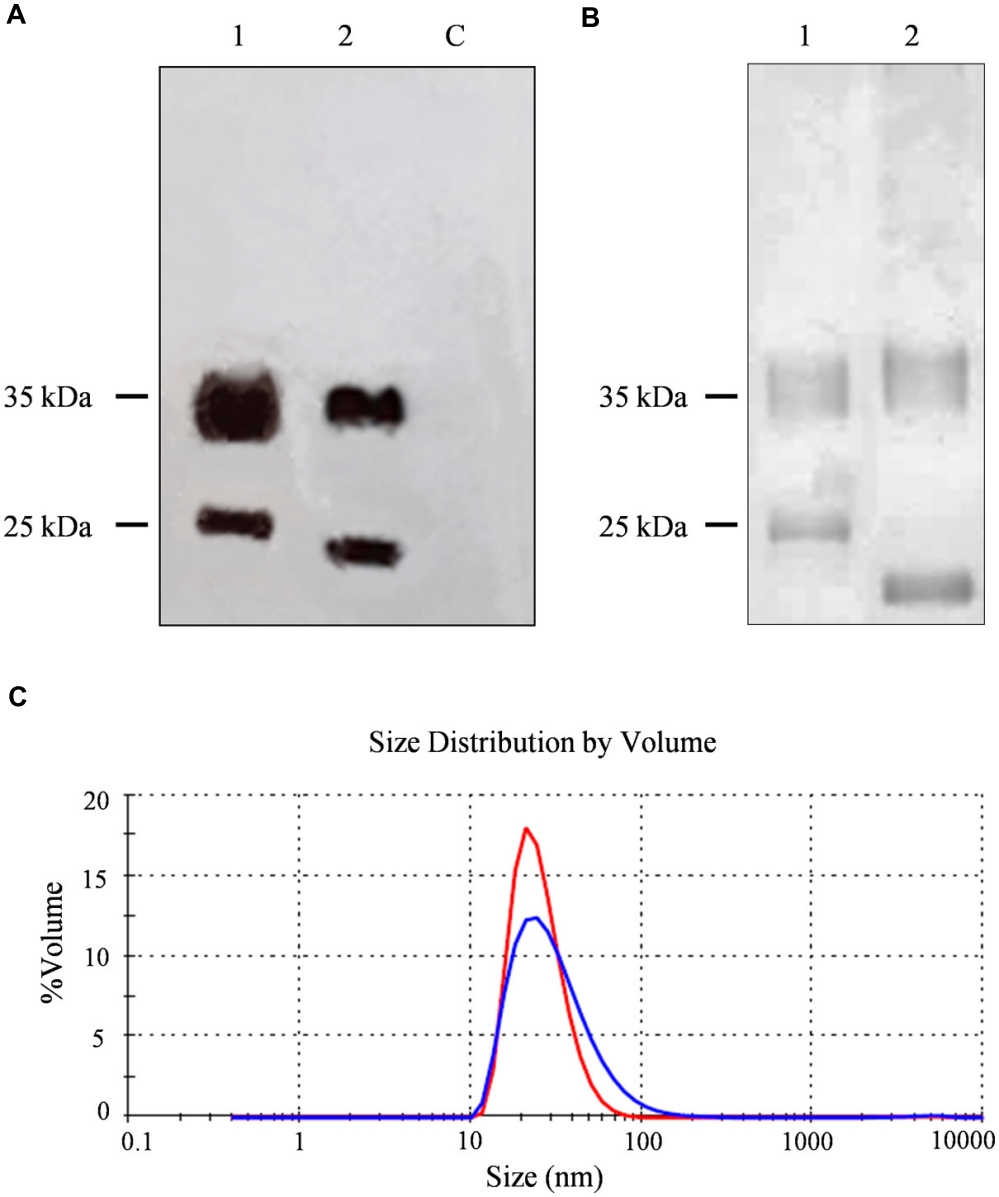
**Analysis and characterization of CPMV eVLPs. (A)** Western blot of *N. benthamiana* leaf TSP extracts, 11 μl per lane separated by SDS-PAGE and detected with an alkaline phosphatase conjugated anti-CPMV CP antibody. C = non-infected leaf extract, 1 = CPMV-lipo leaf extract, and 2 = CPMV control leaf extract. **(B)** Silver staining of 2 μg purified particles separated by SDS-PAGE. 1 = CPMV-lipo particles and 2 = CPMV control particles. Arrows indicate the position of the 25 and 35 kDa molecular markers. **(C)** DLS analysis comparing purified CPMV-lipo (blue line) to unmodified CPMV (red line).

The CPMV-lipo eVLPs were produced using the pEAQ-*HT* system (Sainsbury et al., 2009). The VP60 CP precursor and the 24K protease were expressed on two separate pEAQ constructs allowing the production of eVLPs structurally identical to wild-type CPMV particles (Saunders et al., 2009). The sequence encoding the target peptide was inserted into vector pEAQ*-HT*-VP60, within the βB–βC loop of the small CP. *N. benthamiana* leaves were agroinfiltrated simultaneously with both vectors. Leaf extracts were prepared 6 dpi for analysis by western blot using anti-CPMV antibodies, revealing the presence of specific signals corresponding to the large and small and a molecular weight shift accounting for the modified small CP, thus confirming the insertion of the foreign peptide (**Figure 2A**).

A larger number of plants were infiltrated to obtain leaf material for the purification of unmodified CPMV and CPMV-lipo eVLPs. Clarified leaf protein extracts were processed by anion exchange chromatography column to remove phenolic compounds (Gecchele et al., 2014). The flow through fraction containing the eVLPs was concentrated and then purified by size-exclusion chromatography. We achieved final yields of ∼0.04 mg/g and ∼0.05 mg/g FLW for the modified and unmodified eVLPs, respectively. The purity of the eVLPs was confirmed by SDS-PAGE and silver staining (**Figure 2B**). DLS confirmed the correct assembly of the purified CPMV-lipo eVLPs and revealed that the spherical particles had an outer diameter of ∼28 nm as anticipated (Aljabali et al., 2010), (**Figure 2C**).

### Development of a VNP-based Direct ELISA

The suitability of modified VNPs for the diagnosis of pSjS was tested by comparing ELISAs based on the free synthetic peptide to the different VNP formats, and also by comparing modified and unmodified VNPs, using sera from pSjS patients, healthy controls and patients with other systemic autoimmune diseases (Supplementary Figure S1). Preliminary comparative analysis of the modified and unmodified VNPs allowed us to discard the CPMV platform immediately because the unmodified particles generated a stronger signal than the CPMV-lipo eVLPs and any attempt to reduce this background failed (Supplementary Figure S1B). All further experiments were therefore carried out using the PVX platform.

**FIGURE 3:**
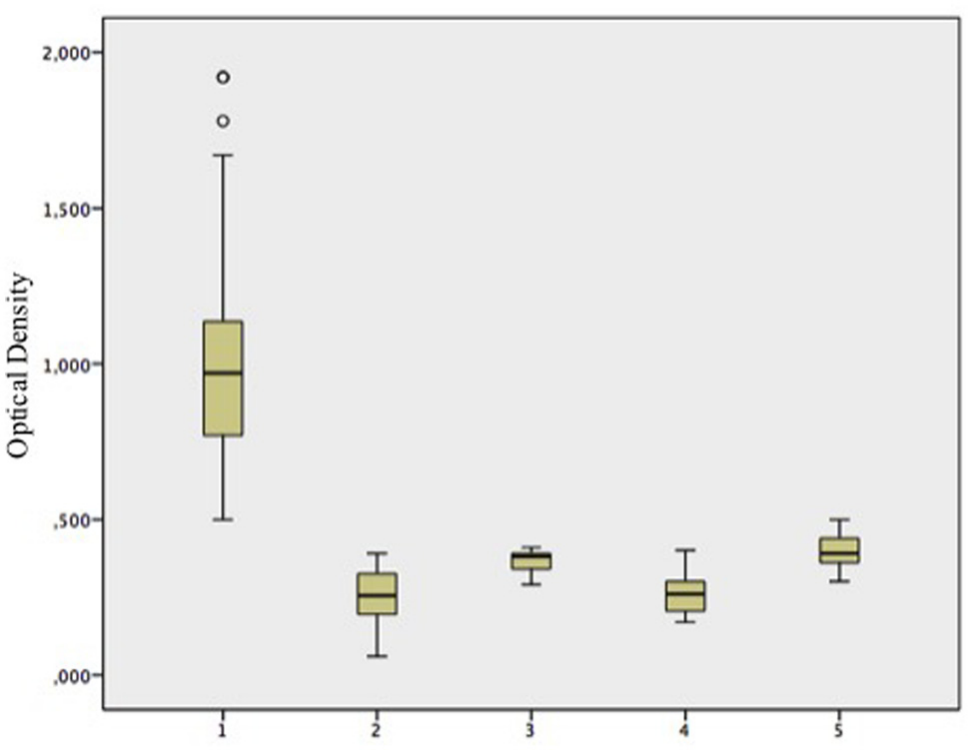
**PVX-lipo ELISA results using sera from different cohorts of patients and controls, expressed in box-plot as mean absorbance (optical density) ± SD.** 1 = pSjS patients (*n* = 91), 2 = healthy donors (*n* = 60), 3 = RA patients (*n* = 20), 4 = SSc patients (*n* = 20), and 5 = SLE patients (*n* = 20).

We compared the PVX platform with the synthetic peptide to evaluate the sensitivity and specificity of each ELISA. We found that 90/91 patients with pSjS were correctly shown to possess serum antibodies against lipocalin using the chimeric PVX particles, whereas 79/91 were identified using the lipocalin peptide alone. These results corresponded to a sensitivity of 86.8% for the synthetic peptide and 98.8% for the PVX-lipo particles (**Table 2**). Neither the synthetic peptide nor the PVX-lipo particles were recognized by sera from healthy donors. The proportion of positive results in the alternative systemic autoimmune disease cohort was fewer than 10% of the SLE patients, corresponding to the occurrence of sSjS, in agreement with our previous data (Suresh et al., 2015), (**Figure 3**; Supplementary Table S1). Autoimmune reactivity against the lipo peptide therefore appears to be largely confined to the pSjS patient population. The specificity of quantitative analysis was 90% for both the PVX-lipo and synthetic lipocalin peptide ELISAs (**Table 2**).

**Table 2:** The sensitivity and specificity (expressed as a percentage) of ELISAs based on the synthetic lipo peptide and PVX-lipo considering all pSjS patients or the pSjS subgroup without ANA as a diagnostic serological marker.

	All pSjS patients		ANA-negative pSjS subgroup	
	Sensitivity	Specificity	Sensitivity	Specificity
Synthetic lipo peptide	86.8	90	75	90
PVX-lipo	98.8	90	98.7	90

We found that sera from the ANA-negative subgroup of SjS patients with diagnostic salivary gland histology (25/91 patients) reacted toward the PVX-lipo particles with a sensitivity of 98.7%, compared to 75% sensitivity in the peptide ELISA. There was also a positive correlation between ANA titers and the ELISA outcome. Patients with an ANA titer exceeding 1:160 produced strong signals in the ELISA (*p* = 0.023), although there was no statistically significant relationship between ANA titers and the presence of anti Ro-SSA or anti La-SSB antibodies. Moreover, there was a strong correlation between the ELISA outcome and exocrine gland symptoms (*p* < 0.001) but not with the involvement of other organs, e.g., pulmonary fibrosis, neurological pathology, or vasculitis. This may reflect the small proportion of patients in our pSjS cohort presenting with these symptoms.

### Stability of the ELISA Test Over the Time

**FIGURE 4:**
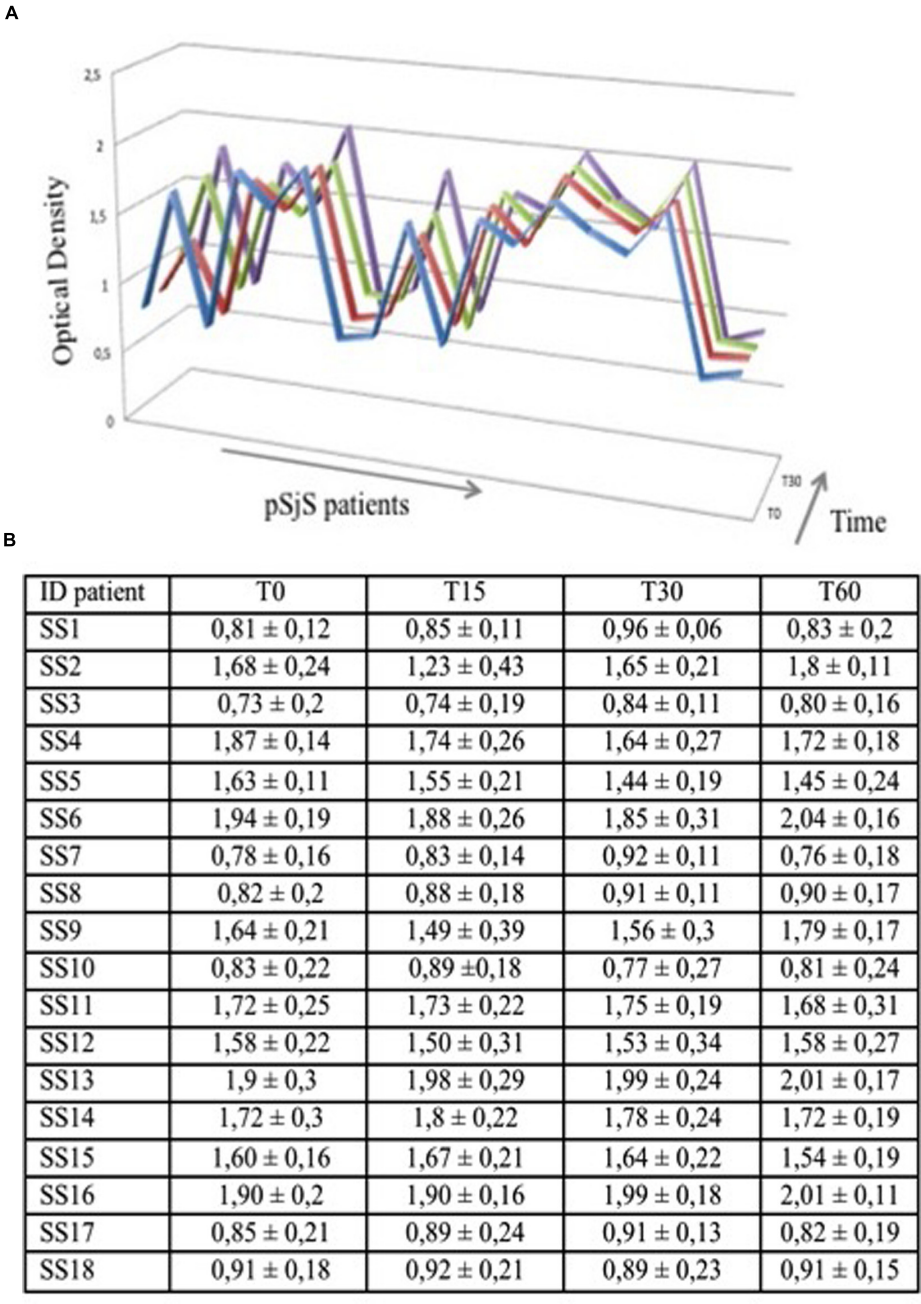
**PVX-lipo ELISA results using sera from a subgroup of 18 pSjS patients at four time points to determine assay stability. (A)** Each serum sample was tested three times at each time point; average values of optical density are graphed on the y axis. Blue = 24 h, red = 15 days, green = 30 days, and purple = 60 days. **(B)** Data set reporting the graphed data in **(A)** for each time point and each patient.

Finally, to demonstrate the stability and reproducibility of the VNP-based ELISA, microtiter plates were coated with PVX-lipo, stored at 4°C and then used after 1, 15, 30, and 60 days to test the sera from 18 SjS patients. The results of these tests did not change regardless of the duration of storage, confirming the stability of the PVX-based ELISA format and the reproducibility of the assay (**Figure 4**).

## Discussion

Primary SjS is a chronic autoimmune disorder characterized by a spectrum of clinical features that are not always manifested in each patient, and that may also be shared with other autoimmune diseases, resulting in delayed and incorrect diagnoses. A key diagnostic criterion is the presence of antinuclear antibodies, but it is not unusual to encounter patients with pSjS-like symptoms lacking this feature given that ANA titers are detected in only 60–85% of patients (Suresh et al., 2015).

In such cases, more invasive tests such as salivary gland and labial biopsies must be used to confirm the diagnosis but on the basis of the negativity of ANA, the salivary gland biopsy may sometimes be excluded, causing a delay or a real misdiagnosis.

Researchers have therefore focused on the development of diagnostic tests for immunodominant autoantigens related to pSjS. Antibodies against SP1, CA6, and PSP have been investigated, but they appear to correlate primarily with symptoms such as dry eyes and mouth and not with the outcome of labial biopsies that provide a definitive pSjS diagnosis (Suresh et al., 2015). The analysis of microRNAs targeting the autoantigens Ro/SSA and La/SSB has suggested that miRNA expression levels could be used to diagnose pSjS, but a reproducible gene expression profile in the disease has not been confirmed (Gourzi et al., 2015).

The screening of a random peptide library with IgG from the sera of pSjS patients identified the immunodominant peptide tear lipocalin, which can distinguish sera from pSjS patients, healthy donors and individuals with other systemic autoimmune disorders when used in an ELISA assay (Navone et al., 2005). However, the sensitivity of diagnostic ELISA test based on synthetic peptides is often reduced by the interaction between the peptide and solid substrate, because the functional epitope can be obstructed. We therefore sought to determine whether the synthetic lipo peptide could be replaced with VNPs displaying the peptide on a CP scaffold.

Chimeric PVX particles were generated by fusing the lipocalin peptide to the N-terminal region of the PVX CP. The recombinant virus induced a systemic infection in *N. benthamiana*, the model host for PVX, and the symptoms were indistinguishable from those caused by the wild-type virus (data not shown). Chimeric PVX particles displaying the lipocalin peptide (PVX-lipo) were genetically stable over three passages of infection in plants (data not shown) and it was therefore possible to propagate the virus and obtain a sufficient mass of infected leaf tissue for the large-scale purification of PVX-lipo particles. The lipocalin peptide meets all the requirements for the formation of stable chimeric PVX particles while preserving virus movement through the plant (Lico et al., 2006), i.e., a length of 12 amino acids, an isoelectric point of 8.75, and the absence of tryptophan residues. The overall architecture of the flexuous chimeric particles was similar to that of wild-type PVX.

*Cowpea mosaic virus* eVLPs displaying the lipocalin peptide were produced by the proteolytic processing of CP precursors in *N. benthamiana* plants as previously described (Saunders et al., 2009). The peptide sequence did not interfere with the ability of the modified CPs to assemble into CPMV-lipo eVLPs, which accumulated to similar levels as the unmodified control eVLPs.

Both VNP systems were tested for their ability to recognize anti-lipocalin autoantibodies present specifically in sera obtained from pSjS patients. These preliminary tests revealed that the CPMV-lipo eVLPs showed the same reactivity as their native counterparts, whereas the PVX-lipo particles reacted specifically to the pSjS patient sera and performed better than the synthetic lipocalin peptide.

The difference in performance between the two VNP platforms may reflect their distinct structures. PVX particles comprise ∼1300 copies of identical CPs arranged in a helical configuration (Atabekov et al., 2007) whereas the icosahedral CPMV particle comprises 60 copies each of the small and large CP subunits, only the former of which is modified, resulting in the display of 60 lipo peptides. The CPMV particles are 28 nm in diameter, compared to PVX with a diameter of 12 nm but a length of ∼550 nm. This means that PVX particles display approximately 10 times as many peptides in the same unit of surface area (PVX = 6.2 × 10^-2^ peptides/nm^2^ and CPMV = 6.09 × 10^-3^ peptides/nm^2^). Furthermore, the rigid icosahedral structure of CPMV and the position of the peptide within the β-loop of the small CP subunit may constrain its ability to be displayed whereas the peptides displayed on the surface of PVX are fused to the N-terminus of the CP, leading to a more flexible and mobile structure maintaining the linear conformation, which correctly promotes antibody recognition.

The ELISA based on the PVX-lipo particles showed greater sensitivity than the corresponding assay with synthetic peptide. This may reflect the relatively flat nature of the synthetic peptide ELISA, with the synthetic peptide passively adsorbed to the two-dimensional polystyrene surface of the microtiter plate, compared to the three-dimensional nature of the VNPs, which form a nanomolecular scaffold that increases the surface area of the plate available for peptide display.

The PVX-based ELISA was also more reproducible than the peptide ELISA (data not shown), reflecting the nature of interaction between the binding reagent and the solid substrate. Synthetic peptides are passively and non-covalently adsorbed to the solid substrate, an interaction which is weak due to the shortness of the peptide, and the structure of the peptide is randomly oriented with respect to the surface plane (Griesmann et al., 1991). In contrast, large and complex multimeric proteins such as VNPs adsorb strongly to the surface, and regardless of orientation, there remain plenty of peptides projecting freely into solution to permit interactions with serum antibodies. This increases the robustness and reproducibility of the ELISA regardless of the chemical structure of the peptide. However, synthetic peptide-based ELISA can be improved by chemical modifications, such as biotinylation, that may help in maintaining peptide conformation and improve their adsorption to solid substrates (Jensen et al., 2000).

The VNP-based ELISA was also remarkably stable. The plate coated with PVX-lipo particles was stored at 4°C for up to 2 months before use without any loss of sensitivity or specificity. This again is likely to reflect the intrinsic characteristics of the modified virus particles which have evolved to be resilient, although the precise basis of their exceptional stability and robustness remains unknown (Hammond and Hull, 1981; Lukashina et al., 2012).

To our knowledge this is the first report in which plant VNPs have been used as a potentially diagnostic assay for a human disease. Our results indicate that plant VNPs can be used to display immunoreactive peptides for the ELISA-based detection of serum autoantibodies and that VNP-based assays are more sensitive than those based on synthetic peptides, probably reflecting their much greater degree of multivalency. Although many different viruses have been used for peptide display, plant viruses are particularly suitable for the manufacture of diagnostic ELISAs, because they can be produced efficiently and inexpensively at high titers in plants, thus making plant suitable for the production of nanostructures that are difficult to express as active assemblies in microbial cells.

## Conclusion

Our data confirm that the autoantigen lipocalin allows the diagnosis of pSjS even in those patients lacking typical disease markers such as antinuclear antibodies and antibodies against Ro/SSA and La/SSB. We have demonstrated that plants may be used to produce nanomaterials suitable for iagnostic assay development, suggesting that the concept we have developed in the context of pSjS could be extended to other diseases.

## Author Contributions

LA and ET developed the research hypothesis, the study design, and drafted the manuscript. MP, CLL, AP, CHL, and EB contributed to the interpretation of data and critically revised the manuscript. MM and RZ prepared and characterized the VNPs. RB and CB performed the ELISAs. Calculation of statistical analysis was performed by ET.

## Conflict of Interest Statement

A patent on the ELISA described in this manuscript was filed on 03/06/2015 at the Italian Patent Office (application 102015000020005).

## Supplementary Material

FIGURE S1 **Comparison of results obtained in ELISA tests using PVX and CPMV as scaffolds for lipo peptide. (A)** PVX-lipo and PVX OD results using sera from a sub-group of 20 SjS patients. Each serum sample was tested three times for each system; average values of optical density are graphed on the *y*-axis. **(B)** CPMV-lipo and CPMV OD results using sera from the same sub-group of 20 SjS patients. Each serum sample was tested three times for each system; average values of optical density are graphed on the *y*-axis. Red line was used to represent VNPs displaying the lipocalin peptide while the blue line represents empty viral scaffolds.
