# Supplementary figures and images for: Plant-Derived Chimeric Virus Particles for the Diagnosis of Primary Sjögren Syndrome
Source: Front Plant Sci. 2015 Dec 1;6:1080. doi: 10.3389/fpls.2015.01080 (PMC4664701; doi:10.3389/fpls.2015.01080)

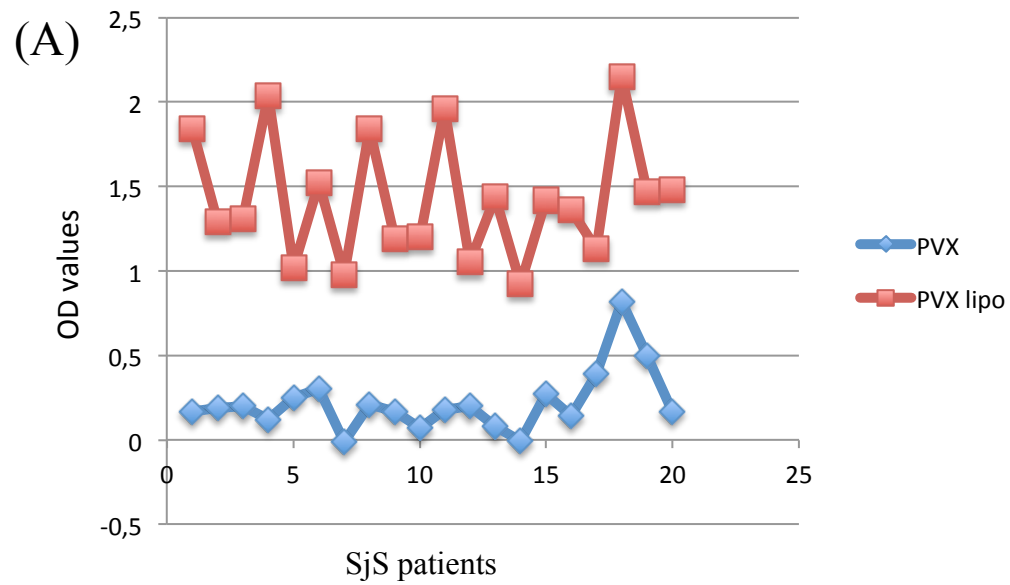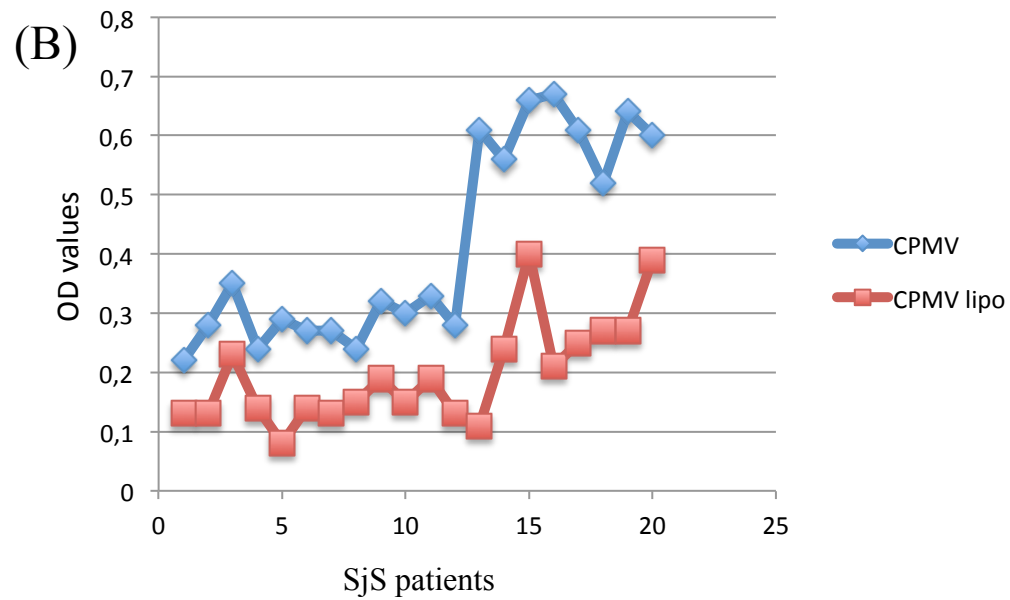

Supplement: FIGURE S1 — Comparison of results obtained in ELISA tests using PVX and CPMV as scaffolds for lipo peptide. (A) PVX-lipo and PVX OD results using sera from a sub-group of 20 SjS patients. Each serum sample was tested three times for each system; average values of optical density are graphed on the y-axis. (B) CPMV-lipo and CPMV OD results using sera from the same sub-group of 20 SjS patients. Each serum sample was tested three times for each system; average values of optical density are graphed on the y-axis. Red line was used to represent VNPs displaying the lipocalin peptide while the blue line represents empty viral scaffolds. [file Image_1.PDF]
