# Supplementary material for: Plant-Derived Chimeric Virus Particles for the Diagnosis of Primary Sjögren Syndrome
Source: Front Plant Sci. 2015 Dec 1;6:1080. doi: 10.3389/fpls.2015.01080 (PMC4664701; doi:10.3389/fpls.2015.01080)
Supplement: Supplementary file 2 [file Table_1.PDF]

Supplementary Table 1: Average OD values in ELISA test for lipocalin.
In column A the OD values of ELISA test with syntethic lipocalin in SjS subjects are reported while in columns B-F the OD values are referred to test with PVX-lipo respectively in SjS patients (B), healthy controls (C), RA subjects (D), SSc patients (E) and SLE patients (F).

	A	B	C	D	E	F
1	0,76	1,67	0,33	0,41	0,26	0,39
2	0,81	1,1	0,19	0,39	0,18	0,41
3	0,72	1,3	0,22	0,36	0,24	0,38
4	0,94	1,92	0,29	0,38	0,29	0,36
5	0,58	0,77	0,31	0,34	0,26	0,31
6	0,16	1,2	0,21	0,29	0,18	0,5
7	0,37	0,99	0,06	0,41	0,24	0,37
8	1,43	1,63	0,23	0,39	0,19	0,38
9	0,96	1,02	0,37	0,36	0,31	0,42
10	1,07	1,13	0,2	0,38	0,34	0,45
11	1,27	1,78	0,32	0,34	0,27	0,48
12	0,76	0,85	0,33	0,29	0,29	0,36
13	0,97	1,36	0,34	0,41	0,38	0,44
14	0,83	0,93	0,24	0,39	0,28	0,47
15	1,09	1,15	0,15	0,36	0,22	0,39
16	1,08	1,22	0,19	0,38	0,17	0,3
17	0,64	0,74	0,21	0,34	0,35	0,35
18	1,24	1,33	0,31	0,29	0,4	0,36
19	0,87	0,97	0,36	0,41	0,23	0,43
20	0,99	1,31	0,28	0,39	0,19	0,44
21	1,02	1,1	0,34			
22	1,05	1,11	0,29			
23	0,73	0,82	0,34			
24	0,75	0,77	0,18			
25	1,07	1,14	0,23			
26	0,85	1,17	0,29			
27	1,03	1,38	0,34			
28	1,02	1,08	0,25			
29	0,94	0,98	0,16			
30	0,68	0,74	0,13			
31	0,54	0,57	0,27			
32	0,6	0,63	0,26			
33	0,52	0,69	0,3			
34	0,82	1,12	0,37			
35	0,9	1,04	0,14			
36	1,02	1,35	0,27			
37	0,91	1,36	0,16			
38	0,72	0,84	0,18			
39	0,92	1,17	0,22			
40	0,82	0,97	0,33			
41	0,01	1,39	0,39			
42	0,01	1,13	0,28			

43	0,63	0,77	0,24
44	0,55	0,65	0,19
45	0,64	0,74	0,33
46	0,7	0,73	0,19
47	0,83	0,98	0,22
48	0,08	0,95	0,29
49	0,01	0,29	0,31
50	0,4	0,45	0,21
51	0,49	0,57	0,06
52	0,97	1,02	0,23
53	0,82	0,9	0,37
54	0,97	1,02	0,2
55	0,6	0,77	0,32
56	0,5	0,61	0,33
57	0,7	0,74	0,34
58	0,68	0,73	0,24
59	0,67	0,99	0,15
60	0,86	0,95	
61	0,2	0,29	
62	0,8	0,87	
63	0,67	0,95	
64	0,79	0,87	
65	1	1,016	
66	0,8	0,9	
67	0,68	0,75	
68	0,58	0,6	
69	0,63	0,7	
70	0,86	0,92	
71	0,7	0,71	
72	0,56	0,98	
73	0,77	0,84	
74	0,6	0,8	
75	0,53	0,61	
76	0,65	0,78	
77	0,6	0,68	
78	0,81	0,97	
79	0,96	1,67	
80	0,91	1,1	
81	1,12	1,3	
82	1,14	1,92	
83	0,68	0,77	
84	1,16	1,2	
85	0,67	0,99	
86	1,03	1,63	
87	0,96	1,02	
88	0,97	1,13	
89	0,79	0,89	

90	0,89	0,95
91	0,78	1,08
